# Advance Care Planning for Care-Dependent Older Persons Living at Home: A Cluster-Randomized Controlled Trial

**DOI:** 10.1093/geroni/igaa057.2301

**Published:** 2020-12-16

**Authors:** Sascha Köpke, Katharina Silies, Rieke Schnakenberg, Änne Kirchner, Juliane Köberlein-Neu, Gabriele Meyer, Falk Hoffmann

**Affiliations:** 1 University of Cologne, Cologne, Germany; 2 University of Lübeck, Lübeck, Schleswig-Holstein, Germany; 3 University of Oldenburg, Oldenburg, Niedersachsen, Germany; 4 Martin Luther University Halle-Wittenberg, Halle (Saale), Sachsen-Anhalt, Germany; 5 Bergische Universität Wuppertal, Wuppertal, Nordrhein-Westfalen, Germany; 6 Carl von Ossietzky University Oldenburg, Oldenburg, Niedersachsen, Germany

## Abstract

Advance Care Planning (ACP) for care-dependent older persons living at home is an important part of care, but remains difficult to implement, mostly due to access barriers. The aim of this trial is to increase patient activation, family communication and surrogate designation through an ACP-intervention delivered by trained nurses to care-dependent clients in their homes. The intervention is evaluated in a cluster-randomised controlled trial in Germany (DRKS00016886). Primary outcome is patient activation (PAM-13); secondary outcomes cover institutionalisation, ACP-engagement and prevalence of ACP-documents. 28 home care services (HCS) with 20 trained nurses and about 340 participants have been included. First results show that patients and caregivers judged the topic and the discussion with trusted persons as important and seized the opportunity for communication. In conclusion, established relationships can be built upon to ensure access to ACP and thus to avoid involuntary treatment in situations of decisional incapacity. Part of a symposium sponsored by Systems Research in Long-Term Care Interest Group.

